# Cardiovascular Risk Awareness Among Adults in the Northern Border Region of Saudi Arabia: A Cross-Sectional Study with Emphasis on Hypertension and Type 2 Diabetes

**DOI:** 10.3390/diseases14070233

**Published:** 2026-06-29

**Authors:** Rehab Abdullah Alanazi, Abir Shiban Alenezi, Raghad Jamal Aldhafeeri, Razan Fawaz Alanazi, Aryam Hussain Alshammari, Ghadah Dhiyab Alanazi, Mohammed Khalaf Alenzi, Areen Amer A. Alenezi, Baraah Abu Alsel, Fathia Ahmed Mersal, Eslam K. Fahmy, Safya E. Esmaeel, Manal S. Fawzy

**Affiliations:** 1 Family and Community Department, Faculty of Medicine, Northern Border University, Arar 91431, Saudi Arabia; dr.rehaby55@gmail.com; 2 College of Medicine, Northern Border University, Arar 91431, Saudi Arabia; st202200401@stu.nbu.edu.sa (A.S.A.); st202200253@stu.nbu.edu.sa (R.J.A.); st202200222@stu.nbu.edu.sa (R.F.A.); st202200216@stu.nbu.edu.sa (A.H.A.); st202200212@stu.nbu.edu.sa (G.D.A.); st202200307@stu.nbu.edu.sa (M.K.A.); st202000125@stu.nbu.edu.sa (A.A.A.A.); 3 Medical Sciences & Preparatory Year Department, North Private College of Nursing, Arar 73244, Saudi Arabia; baraahsel@nec.edu.sa; 4 Public Health Nursing Department, College of Nursing, Northern Border University, Arar 91431, Saudi Arabia; fathia.hassan@nbu.edu.sa; 5 Department of Physiology, College of Medicine, Northern Border University, Arar 91431, Saudi Arabia; eslam.kamal@nbu.edu.sa (E.K.F.); safya.ebraheem@nbu.edu.sa (S.E.E.); 6 Center for Health Research, Northern Border University, Arar 73213, Saudi Arabia

**Keywords:** type 2 diabetes, cardiovascular risk, hypertension, knowledge, heart disease, dyslipidemia, risk factors, Saudi Arabia

## Abstract

Background/Objectives: Cardiovascular diseases (CVDs) remain the leading cause of mortality worldwide. Individuals with hypertension (HTN) and type 2 diabetes mellitus (T2DM) are at particularly high risk; however, awareness of cardiovascular risk within the broader community and among high-risk subgroups remains suboptimal. This study aimed to assess CVD risk awareness and its correlates among adults in the Northern Border Region of Saudi Arabia, with a particular focus on individuals with HTN and/or T2DM. Methods: A descriptive, quantitative, cross-sectional survey was conducted among adults aged 18–50 years residing in the Northern Border Region of Saudi Arabia. An anonymous online questionnaire was distributed via social media between October 2025 and January 2026. The survey incorporated a 22-item scale assessing HTN knowledge, T2DM knowledge, and CVD risk awareness (Heart Disease Fact items). Participants were categorized according to self-reported diagnosis (no diagnosis, HTN, T2DM, or both). Descriptive statistics, non-parametric tests, and multivariable regression analyses were used to evaluate knowledge scores and predictors of CVD risk awareness. Results: A total of 458 participants completed the survey. Overall knowledge was moderate (mean 12.40/22; 56.4%), with relatively higher scores for HTN (73.2%) and T2DM knowledge (68.8%), but markedly lower CVD risk awareness (30.8%). Fewer than half of participants correctly answered key CVD items, particularly those related to asymptomatic disease progression and lipid (HDL) concepts. Only 21.6% achieved good awareness (≥75%). In multivariable analyses, higher educational level, positive family history of cardiometabolic disease, and the presence of HTN and/or T2DM were independent predictors of higher awareness. Conclusions: CVD risk awareness is suboptimal among adults in the Northern Border Region, including those with established HTN and T2DM. The observed gap between disease-specific knowledge and CVD risk awareness highlights the need for targeted, structured risk communication strategies in primary care, particularly for individuals with lower educational attainment and no family history of CVD.

## 1. Introduction

Cardiovascular diseases (CVDs) are the leading cause of death worldwide and represent a major public health challenge across all regions [1]. Among non-communicable diseases, diabetes mellitus and arterial hypertension are two of the most prevalent and impactful conditions [2]. An estimated 537 million people are currently living with diabetes globally, the majority of whom have type 2 diabetes mellitus (T2DM), which accounts for roughly 90% of all diabetes cases [3]. Well-established risk factors for CVD include elevated blood pressure, diabetes, dyslipidemia, central obesity, and other features of the metabolic syndrome [4]. A large proportion of CVD- and diabetes-related deaths occur in low- and middle-income countries, where the burden of diabetes and hypertension is rapidly increasing, and health systems may be less equipped to respond [5].

Both diabetes and hypertension substantially increase the risk of microvascular complications such as nephropathy and retinopathy, as well as macrovascular CVD and all-cause mortality [6]. Global estimates suggest that more than one billion adults have hypertension, and hundreds of millions are living with diabetes, with these numbers projected to rise markedly in the coming decades, particularly in developing countries [7]. Data from the American Heart Association indicate that the majority of older adults with diabetes will ultimately die from a cardiovascular cause, underlining the tight link between diabetes and CVD. In individuals with T2DM, peripheral arterial disease and heart failure often represent early clinical manifestations of CVD [8].

In Saudi Arabia and across the Gulf region, rapid urbanization, lifestyle changes, and population aging have contributed to a high and rising prevalence of hypertension, T2DM, obesity, and dyslipidemia [9]. National and regional surveys have consistently documented substantial levels of these cardiometabolic risk factors, with many individuals remaining undiagnosed or suboptimally controlled for blood pressure, glycemic levels, and lipid profiles despite ongoing screening and health promotion efforts [10]. CVD is a leading cause of morbidity and mortality in the country, and chronic disease prevention has become a major priority within Saudi health strategies and Vision 2030 initiatives [11]. Within this context, understanding cardiovascular risk awareness among high-risk groups is essential to inform culturally appropriate, region-specific interventions [12].

Effective prevention of CVD in individuals with diabetes and hypertension requires comprehensive management of modifiable risk factors, including glycemic control, blood pressure, and lipid regulation, smoking cessation, weight management, and lifestyle modification [13,14,15]. Although these strategies are well established, their effectiveness is strongly influenced by patients’ awareness and perception of their own cardiovascular risk [16]. Suboptimal awareness has been linked to poor adherence to therapy, delayed healthcare utilization, and inadequate engagement in preventive behaviors [17].

Despite the growing body of literature on CVD risk awareness, important gaps remain. Most available studies have been conducted either in the general population or in primary care settings without focusing specifically on high-risk groups with established hypertension and T2DM [18,19,20]. In Saudi Arabia, existing research has largely emphasized the prevalence and control of cardiometabolic risk factors, with limited data addressing patients’ awareness and perception of cardiovascular risk, particularly at the regional level [21,22,23]. Moreover, the Northern Border Region remains underrepresented in national research, despite evidence indicating a substantial burden of chronic diseases in this population [21]. To date, there is a lack of region-specific data evaluating how individuals with coexisting hypertension and T2DM perceive their cardiovascular risk and which factors influence this awareness.

Therefore, the present study aims to assess cardiovascular risk awareness among adults in the Northern Border Region of Saudi Arabia, with a particular focus on individuals with hypertension and/or T2DM. In addition, the study seeks to identify sociodemographic and clinical factors associated with awareness levels. By examining both the general population and high-risk subgroups within a relatively understudied region, this study provides context-specific evidence to inform targeted educational and preventive strategies in primary healthcare settings.

## 2. Materials and Methods

### 2.1. Study Design and Setting

This study employed a descriptive, quantitative, cross-sectional survey design to assess cardiovascular risk awareness among adults, with a particular focus on individuals with type 2 diabetes mellitus (T2DM) and/or hypertension. Convenience sampling was used to recruit eligible participants residing in the Northern Border Region of Saudi Arabia. Data were collected over a predefined period, from 27 October 2025 to 5 January 2026, using an anonymous, self-administered online questionnaire distributed through social media platforms.

### 2.2. Population and Participants

The study targeted adults aged 18–50 years, both sexes, residing in the Northern Border Region of Saudi Arabia. Participants were recruited through a convenience sampling approach via an online survey distributed on social media platforms. Individuals with and without a self-reported diagnosis of T2DM and/or hypertension were eligible to participate.

For analysis, respondents were categorized by self-reported clinical status into four groups: no diagnosis, hypertension only, T2DM only, and both conditions. Exclusion criteria included residence outside the Northern Border Region, age under 18 years, diagnosis of type 1 diabetes, gestational diabetes, and refusal to participate in the survey. Only respondents who completed the core knowledge items were included in the final analysis.

### 2.3. Data Collection Tool

Data were collected using a structured, self-administered questionnaire adapted from previously validated instruments [24,25]. The tool comprised sections on sociodemographic characteristics, clinical status, hypertension-related details, diabetes-related details, and cardiovascular disease knowledge based on Heart Disease Fact questions (Supplementary File S1). In total, 22 items contributed to the knowledge assessment and were organized into three subscales: hypertension knowledge (7 items), diabetes knowledge (7 items), and cardiovascular disease (CVD) awareness (8 items).

Sociodemographic variables included age group, sex, marital status, educational level, employment, diagnostic status, family history of CVD, and selected lifestyle factors. The hypertension-related section captured the duration of diagnosis and treatment, as well as knowledge items on complications, symptoms, normal blood pressure levels, and the roles of obesity and physical activity in blood pressure control. The diabetes-related section included duration of diabetes, treatment regimen, monitoring practices, and knowledge items regarding symptoms, glycemic control, obesity, and diabetes-related complications. The CVD component comprised Heart Disease Fact items that addressed awareness of asymptomatic disease, the impact of sex and family history on CVD risk, and understanding of “good” and “bad” cholesterol. This 22-item knowledge scale can rapidly identify domain-specific gaps in HTN, T2DM, and CVD risk awareness, making it a potentially useful brief screening tool in clinical and public health settings.

The CVD awareness items were adapted from the Heart Disease Fact Questionnaire and related tools, which have been used in diabetic and primary care populations and have demonstrated acceptable reliability and validity in previous studies. In this study, the eight-item CVD subscale was intentionally designed to capture several heterogeneous but clinically salient aspects of risk, silent/asymptomatic CVD, lipid (HDL/LDL) targets, and the compounded risk when HTN and T2DM coexist, rather than a narrow, homogeneous construct. Consequently, lower internal consistency is not unexpected and reflects the content’s multidimensional nature. The subscale nonetheless showed coherent associations with education, family history, diagnosis status, and HTN/T2DM knowledge, supporting its suitability as a brief indicator of key CVD risk awareness gaps in this population [26,27,28].

### 2.4. Sample Size

Given the non-probability online recruitment strategy, the sample size was calculated using an epidemiological approach for cross-sectional studies. Assuming a 95% confidence level, a 5% margin of error, and an expected frequency of 50% (to yield the maximum sample size in the absence of prior estimates), the initial required sample size was 384 participants.

To account for the non-random sampling design, a design effect of 1.15 was applied, increasing the sample size to 442 participants. An additional 10% was added to account for incomplete or unusable responses, resulting in a final target sample size of 485 participants.

The study ultimately included 458 complete responses for analysis. Given the convenience sampling approach, the achieved sample size should be interpreted in terms of statistical precision rather than population representativeness, and this limitation is acknowledged.

### 2.5. Reliability and Validity

The internal consistency of the 22-item total knowledge scale and its three subscales (HTN knowledge, DM knowledge, CVD risk awareness) was evaluated using Cronbach’s α and split-half reliability coefficients. It was acceptable (Cronbach’s α = 0.695; 95% CI: 0.653–0.734). The hypertension knowledge subscale demonstrated good reliability (α = 0.845; 95% CI: 0.822–0.865), as did the diabetes knowledge subscale (α = 0.850; 95% CI: 0.828–0.870). In contrast, the CVD risk awareness subscale showed questionable internal consistency (α = 0.565; 95% CI: 0.502–0.623), suggesting some heterogeneity in the construct.

Item-total correlation analysis indicated that all eight CVD risk awareness items had correlations below the commonly used 0.30 threshold (range: 0.117–0.198), indicating limited discrimination. By comparison, five of seven hypertension items and six of seven diabetes items exceeded this threshold. Split-half reliability coefficients were consistent with the Cronbach’s α values (Total scale: 0.761; HTN: 0.827; DM: 0.827; CVD: 0.560), supporting the overall reliability of the core knowledge subscales.

### 2.6. Statistical Analysis

All analyses were performed using SPSS version 26. Categorical variables were summarized as frequencies and percentages. Continuous score variables were assessed for distributional assumptions using both the Shapiro–Wilk test and graphical inspection of histograms and Q-Q plots. Because the score distributions were not normal, non-parametric methods were used for between-group comparisons. Accordingly, the Mann–Whitney U test was used for two-group comparisons and the Kruskal–Wallis test for comparisons involving more than two groups. Percentage scores on the 22-item knowledge scale were categorized into three awareness levels for ordinal analyses: poor (<50%), moderate (50–74%), and good (≥75%). These cutoffs were informed by previous cardiovascular knowledge and Heart Disease Fact Questionnaire-based studies, which typically define scores below 50% as low/poor knowledge, intermediate ranges around 50–70% or 50–74% as moderate/fair knowledge, and scores above 70–75% as good or optimal. In this study, <50% was considered clearly inadequate, whereas ≥75% was chosen to reflect a high level of awareness across the HTN, T2DM, and CVD domains. Categorical distributions of awareness levels were examined using chi-square tests [29,30,31]. Effect sizes were reported, where applicable, using Cohen’s d, eta-squared (η^2^), and Cramér’s V.

Four regression models were fitted to examine factors associated with cardiovascular disease (CVD) awareness. Ordinary least squares (OLS) linear regression was used to model the total knowledge score, ordinal logistic regression was used to model the three-level awareness outcome, and binary logistic regression was used to model good awareness (≥75%). In addition, a cross-domain OLS model was developed to assess the extent to which hypertension and diabetes knowledge scores, along with selected demographic variables, predicted CVD risk awareness.

Multivariable modeling was conducted using a forced-entry (Enter) approach, in which predictor variables were included simultaneously based on their a priori clinical and conceptual relevance rather than automated stepwise procedures. Multicollinearity diagnostics were performed using variance inflation factors (VIF) and condition indices. Evidence of severe collinearity was observed for age (VIF = 11.3), with moderate collinearity detected for education (VIF = 7.8) and weight category (VIF = 5.7). Condition indices reached 47.3, accompanied by high proportions of variance decomposition for age and education. Standard diagnostic checks were reviewed to ensure model validity before interpretation of the results. These variables were retained due to their conceptual relevance, stability of estimated effects in sensitivity analyses, and acceptable VIF thresholds for epidemiologic adjustment models [32,33].

Model diagnostics indicated acceptable residual independence (Durbin–Watson = 1.817) and no significant heteroscedasticity (Breusch–Pagan *p* = 0.093). Mild non-normality of residuals was considered acceptable given the large sample size. Model performance was evaluated using R^2^ and pseudo-R^2^ statistics, and the 5-fold cross-validated area under the receiver operating characteristic curve (AUC) for the logistic model (AUC = 0.644). All analyses were performed using SPSS version 26 (IBM Corp., Armonk, NY, USA), and a *p*-value < 0.05 was considered statistically significant. GAAbstract (https://gaabstract.com/), accessed on 27 May 2026, was used to generate the initial draft of Figure 4 and the graphical abstract from the edited text. The authors have reviewed and edited the output and take full responsibility for the content of this outcome.

### 2.7. Ethical Considerations

Participant anonymity and confidentiality were strictly maintained throughout the study. No names or personally identifying information were collected, enabling participants to respond honestly without concern about potential effects on their personal or professional lives. Informed consent was obtained electronically from all participants before they accessed the online questionnaire. The invitation clearly explained the study purpose, the voluntary nature of participation, and the right to decline or withdraw at any time without any consequences. All responses were stored securely and used solely for research purposes. Ethical approval for the study was obtained from the local bioethics committee of North Private College of Nursing (Protocol number: 22092025-71).

## 3. Results

### 3.1. Study Sample and Participant Characteristics

A total of 458 participants were included in the analysis. The sample was predominantly female (73.1%, *n* = 335), with males comprising 26.9% (*n* = 123). Age distribution was bimodal, with 30.1% aged 18–25 years and 30.4% aged 36–46 years, while those aged 46+ accounted for 26.0% of the sample. Educational attainment was generally high: 72.3% had a university degree or higher, 20.1% had secondary education, 4.8% had basic education, and 2.8% were illiterate.

Clinically, most participants reported no diagnosis of hypertension (HTN) or type 2 diabetes mellitus (T2DM) (63.8%, *n* = 292). In contrast, 11.4% reported HTN only (*n* = 52), 12.0% T2DM only (*n* = 55), and 12.9% comorbid HTN + T2DM (*n* = 59). A positive family history of HTN and/or DM was reported by 65.1% (*n* = 298). Most participants were non-smokers (87.1%), and nearly two-thirds (64.0%) reported low physical activity (Table 1).

### 3.2. Knowledge Domains and Overall Awareness Classification

Participants’ performance differed markedly across the three knowledge domains (Table 2). HTN knowledge was highest (mean = 5.12, SD = 1.40; 73.2%), followed by DM knowledge (mean = 4.81, SD = 1.60; 68.8%). In contrast, CVD risk awareness was substantially lower (mean = 2.46, SD = 1.89; 30.8%), indicating a pronounced domain-specific gap in CVD literacy, despite comparatively stronger knowledge of HTN and DM.

The mean total knowledge score was 12.40 (SD = 4.78) out of 22, corresponding to 56.4%. Based on prespecified percentage cutoffs, 33.2% of participants were classified as having poor awareness (<50%), 45.2% as having moderate awareness (50–74%), and 21.6% as having good awareness (≥75%) (Table 2).

Item-level analysis supported this pattern. HTN knowledge items were consistently above the 50% correctness threshold (71.2–85.4%), and most DM items exceeded 50% (65.4–80.8%), with one exception (“DM patients prone to infection”, 40.6%). By comparison, all CVD risk awareness items were well below 50% (9.8–28.6%), with particularly low correct responses to HDL-related knowledge and to the recognition that CVD may be asymptomatic, highlighting critical gaps in risk-related understanding (Supplementary Figure S1).

### 3.3. Distribution of Awareness Categories by Diagnosis Status, Sex, and Family History

**Figure 1:**
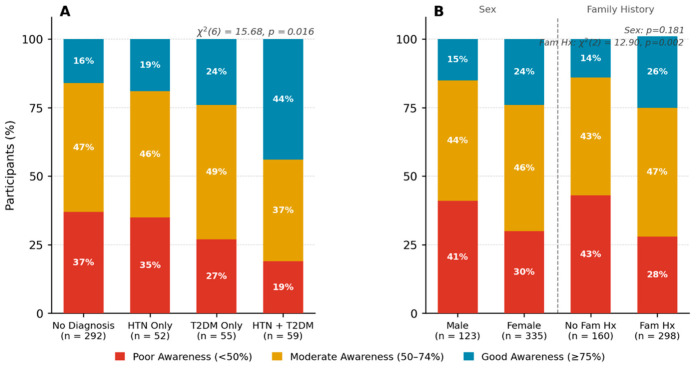
Distribution of CVD risk awareness levels by key characteristics. (**A**) Distribution of awareness levels by diagnosis status (no diagnosis, hypertension only, type 2 diabetes only, and combined hypertension and type 2 diabetes). Stacked bars show proportions of poor (<50%), moderate (50–74%), and good (≥75%) awareness. Differences between groups were statistically significant (χ^2^(6) = 15.68, *p* = 0.016). (**B**) Distribution of awareness levels by sex and family history of CVD. Stacked bars show proportions of poor, moderate, and good awareness. Differences by family history were statistically significant (χ^2^(2) = 12.90, *p* = 0.002), whereas differences by sex were not statistically significant (*p* = 0.181).

Awareness category distributions varied significantly by diagnosis status (Figure 1A; χ^2^(6) = 15.68, *p* = 0.016). Participants with comorbid HTN + T2DM had the highest proportion of good awareness (44%) and the lowest proportion of poor awareness (19%), compared with those reporting no diagnosis (good: 16%; poor: 37%).

In contrast, awareness distributions did not differ significantly by sex (*p* = 0.181), although females showed a higher proportion of good awareness than males (24% vs. 15%) (Figure 1B). Family history was associated with awareness (χ^2^(2) = 12.90, *p* = 0.002): participants with a positive family history had a higher proportion of good awareness (26% vs. 14%) and a lower proportion of poor awareness (28% vs. 43%) compared with those without a family history.

### 3.4. Between-Group Differences in Total Knowledge Score

Univariate analyses identified several significant correlates of total knowledge score (Table 3). Females scored higher than males (Mann–Whitney U = 17,234, *p* = 0.022, Cohen’s d = 0.21). Total knowledge differed across age groups (Kruskal–Wallis H = 10.8, *p* = 0.013, η^2^ = 0.07) and education levels (H = 14.9, *p* = 0.002, η^2^ = 0.09). Family history showed the largest effect: participants with a positive family history had significantly higher knowledge (U = 17,543, *p* < 0.001, d = 0.48).

Diagnosis status was also associated with knowledge (H = 10.0, *p* = 0.018, η^2^ = 0.06), with higher scores observed among participants with a reported diagnosis. Geographic location and smoking status were associated with smaller but statistically significant differences in total knowledge scores (Table 3).

### 3.5. Multivariable Predictors of CVD Risk Awareness

**Figure 2:**
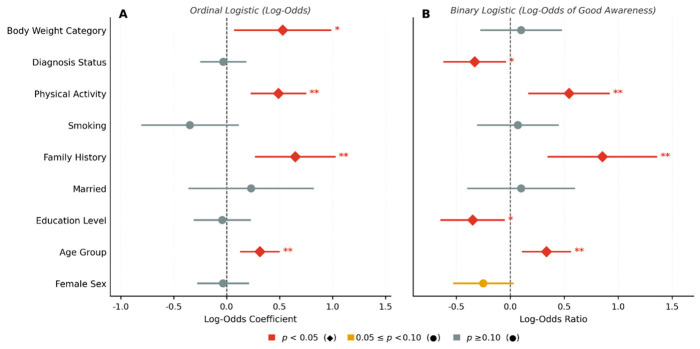
Multivariable regression forest plots. (**A**) Ordinal logistic regression model for CVD risk awareness (log-odds). Points represent estimated coefficients and horizontal lines indicate corresponding 95% confidence intervals. Positive coefficients indicate higher odds of greater awareness levels. (**B**) Binary logistic regression model for good awareness (≥75%) (log-odds). Points represent estimated log-odds ratios, and horizontal lines indicate 95% confidence intervals. Positive values indicate a higher likelihood of good awareness. In both panels, the dashed vertical line represents the null effect. Statistical significance is denoted as * *p* < 0.05 and ** *p* < 0.01.

Two multivariable regression models were estimated to identify independent predictors of CVD risk awareness (Table 4; Figure 2). In the ordinal logistic model predicting progression to higher awareness categories (poor → moderate → good), female sex (log-OR = 0.529, SE = 0.235, *p* = 0.024), higher education level (log-OR = 0.489, SE = 0.134, *p* < 0.001), positive family history (log-OR = 0.647, SE = 0.195, *p* = 0.001), and diagnosis status (log-OR = 0.313, SE = 0.096, *p* = 0.001) were independently associated with higher awareness. Age, marital status, smoking, physical activity, and body weight were not significant in the adjusted ordinal model.

In the binary logistic model predicting good awareness (≥75%), education (OR = 1.72, 95% CI: 1.18–2.52; *p* = 0.005), family history (OR = 2.35, 95% CI: 1.41–3.90; *p* < 0.001), and diagnosis status (OR = 1.40, 95% CI: 1.11–1.76; *p* = 0.004) remained independent predictors of achieving good awareness (Table 4; Figure 2). Non-significant variables from the ordinal model were omitted from the final binary model presentation for brevity.

### 3.6. Predicted Probabilities and Cross-Domain Associations

**Figure 3:**
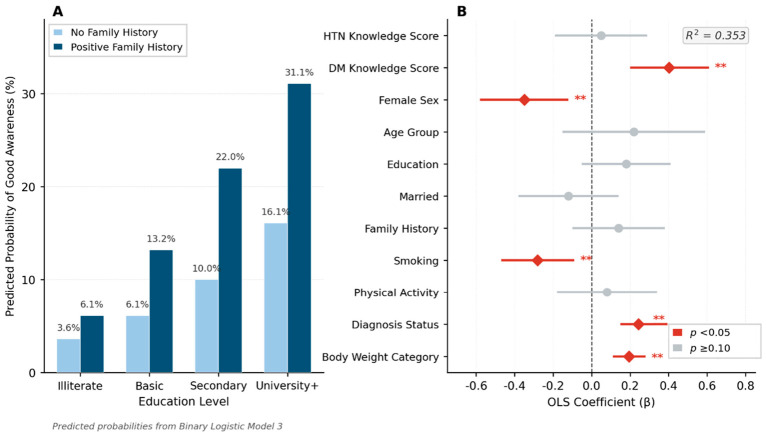
Associations between education, family history, and cardiovascular disease (CVD) awareness. (**A**) Predicted probability of good awareness (≥75%) by education level and family history. Probabilities increase with higher education levels and are consistently higher among individuals with a positive family history than among those without. (**B**) Ordinary least squares (OLS) regression coefficients (β) for predictors of CVD risk awareness. Estimates are shown with confidence intervals; statistically significant predictors are indicated (** *p* < 0.01). Model fit: R^2^ = 0.353.

Model-derived predicted probabilities illustrated a graded increase in the likelihood of good awareness with higher education (Figure 3A). Across all educational strata, participants with a positive family history had higher predicted probabilities than those without one. For example, the predicted probability of good awareness increased from 3.6% (no family history) to 6.1% (positive family history) among illiterate participants, and from 16.1% to 31.1% among those with university education or higher.

In the cross-domain OLS model, both HTN knowledge (β = 0.194, *p* < 0.001) and DM knowledge (β = 0.244, *p* < 0.001) were positively associated with CVD risk awareness, together explaining 35.3% of the variance (R^2^ = 0.353; Figure 3B). After adjustment for domain knowledge, age (β = −0.281, *p* = 0.003) and physical activity (β = −0.349, *p* = 0.003) were inversely associated with CVD risk awareness, indicating an inverse association between age, physical activity, and CVD risk awareness in this sample. Because the inverse association between physical activity and CVD risk awareness was unexpected, sensitivity analyses were undertaken. When physical activity was modeled as an ordinal three-level variable in the adjusted model, higher physical activity remained inversely associated with CVD risk awareness (β = −0.29, 95% CI −0.57 to −0.01; *p* = 0.046). However, the association was attenuated and no longer statistically significant when the analysis was restricted to respondents with HTN and/or T2DM only (β = −0.34, 95% CI −0.81 to 0.13; *p* = 0.158) and when physical activity was dichotomized as low versus at least weekly activity (β = −0.30, 95% CI −0.67 to 0.08; *p* = 0.122). In the binary logistic model for good awareness, greater physical activity remained associated with lower odds of good awareness (OR = 0.52, 95% CI 0.29–0.94; *p* = 0.030).

## 4. Discussion

Cardiovascular disease remains the leading cause of death globally, with HTN and T2DM as major, synergistic risk factors [34]. This study examined awareness of CVD risk among adults in the Northern Border Region of Saudi Arabia, with particular attention to those with HTN and/or T2DM, and identified sociodemographic and clinical correlates of awareness. The findings reveal a clear domain-specific mismatch: participants showed relatively high knowledge regarding HTN (73.2%) and T2DM (68.8%), but markedly low awareness of CVD risk (30.8%). This pattern mirrors previous work reporting poor knowledge of heart disease risk among patients with diabetes, despite a reasonable understanding of diabetes itself.

The large deficit in CVD risk awareness contrasts with studies from other settings that have documented higher cardiovascular risk knowledge in patients with cardiometabolic disease [35,36]. The current results, therefore, suggest that clinical encounters and public health campaigns in this region may have conveyed core information about primary diagnoses more effectively than about the cumulative, often asymptomatic nature of macrovascular complications. However, because the study did not directly assess the content or quality of clinical counseling or public health messaging, this interpretation should be viewed as a tentative explanation rather than a definitive conclusion. In line with prior reports, participants appeared to understand immediate disease management actions (e.g., medication use, glucose monitoring) but lacked insight into subclinical atherogenesis and the silent presentation of ischemic events [24,37,38,39].

### 4.1. Domain-Specific Knowledge Gaps

The domain-specific analyses underscore this gap. Item-level performance was strong for key behavioral and self-care items, such as the role of regular exercise in blood pressure control (85.4%) and diabetic foot care (80.8%), and largely satisfactory for most diabetes items. In sharp contrast, all CVD risk awareness items fell below 50% correctness, with some below 15%. Only 28.6% recognized that CVD can progress asymptomatically, and fewer than one in six participants correctly identified HDL-related protective concepts and risk thresholds. This pattern indicates that many participants conceptualize HTN and T2DM as isolated conditions rather than components of an integrated cardiometabolic risk continuum. Similar misconceptions have been described elsewhere, where individuals with T2DM underestimated their CVD risk or were unaware of the strength of the T2DM–CVD link [40].

These findings support the argument that CVD literacy is not a single construct but a multi-faceted one, encompassing symptom knowledge, risk factor understanding, lipid and blood pressure targets, and the concept of absolute/global risk [41]. Aggregated total scores may therefore obscure critical thematic deficits. In this cohort, the major “blind spot” was not disease presence but disease trajectory, especially the idea that serious CVD can develop and progress without overt symptoms.

### 4.2. Sociodemographic and Clinical Correlates

Sociodemographic and clinical variables showed meaningful associations with knowledge and awareness. Total knowledge scores varied significantly by sex, age, education, diagnosis status, and family history, consistent with previous evidence that sociodemographic and clinical context influence cardiovascular literacy [42,43,44,45]. Females had higher total knowledge and a higher proportion of good awareness than males, although sex differences in awareness categories were not significant in bivariate analyses. After adjustment, however, females emerged as an independent predictor of higher awareness in the ordinal logistic model. This differs from some previous studies in which hypertensive women had similar or lower CVD risk perception compared with men [46,47,48]. Cultural, educational, or health service utilization patterns in the Northern Border Region may partly explain this discrepancy.

Education showed a graded association with awareness in both ordinal and binary models. Individuals with higher education were more likely to occupy higher awareness categories and to achieve good awareness (≥75%). This is consistent with prior work suggesting that higher educational attainment supports better comprehension and critical appraisal of complex health information, including the multi-system interactions characteristic of “cardio-diabetes” [49,50]. Similarly, a positive family history of HTN/DM substantially increased the odds of good awareness, suggesting that direct exposure to CVD morbidity or mortality within the family may act as a powerful driver of risk recognition beyond routine counseling [51].

Among the diagnostic subgroups, participants with comorbid HTN and T2DM showed the most favorable awareness profile, with the highest proportion of good awareness and the lowest proportion of poor awareness. This pattern likely reflects accumulated clinical exposure and repeated contact with healthcare providers [52]. However, the overall level of CVD risk awareness in this subgroup was still far from optimal, indicating that even among the most clinically engaged patients, critical aspects of risk communication remain incomplete.

### 4.3. Cross-Domain Associations and Behavioral Interpretations

The cross-domain regression model showed that both HTN and DM knowledge were positively associated with CVD risk awareness, jointly accounting for over one-third of the variance in CVD risk awareness scores. This confirms that strengthening disease-specific knowledge can improve risk awareness, though it is not sufficient on its own. The inverse association between age and CVD risk awareness may reflect reliance on older health beliefs, reduced exposure to contemporary risk communication, or fatigue with complex medical information among older adults [24,53,54].

The observed inverse relationship between physical activity and CVD risk awareness is noteworthy but should be interpreted with caution. One plausible explanation is the presence of a “healthy user” effect or optimistic bias, whereby physically active individuals may perceive themselves as inherently protected and consequently pay less attention to information on subclinical risk markers, such as lipid profiles or blood pressure targets [41]. However, this interpretation must be tempered by the instability of the finding. Although the inverse association was evident in the primary model, it was attenuated in sensitivity analyses, suggesting that it may reflect residual confounding, subgroup instability, or imprecision in the self-reported physical activity measure rather than a consistent underlying relationship.

Accordingly, this finding should be considered hypothesis-generating. It nevertheless underscores an important public health message: while physical activity confers substantial cardiovascular benefits, it does not eliminate the need for regular risk assessment and optimal control of hypertension and type 2 diabetes [42]. Further research using diagnosis-confirmed samples and more granular, objective measures of physical activity is warranted to clarify the nature of this association.

### 4.4. Implications for Saudi Primary Care

**Figure 4:**
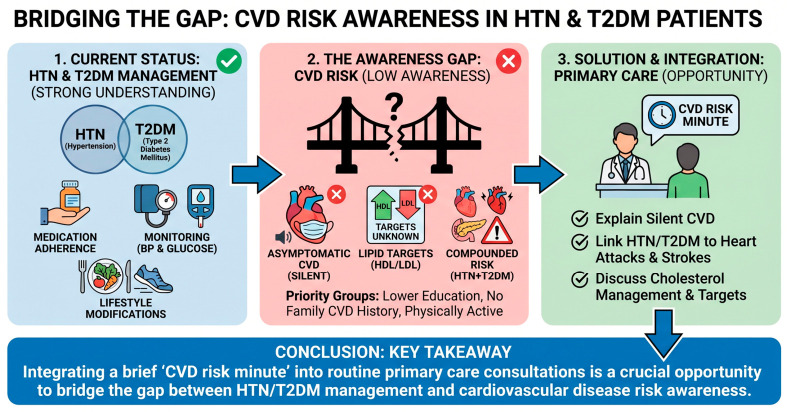
Conceptual pathway linking hypertension (HTN) and type 2 diabetes mellitus (T2DM) management to cardiovascular disease (CVD) risk awareness and primary care intervention among patients with HTN and/or T2DM. The schematic summarizes three stages: (1) the current status, in which patients with HTN and T2DM typically show good understanding of disease management, including medication adherence, blood pressure and glucose monitoring, and lifestyle modification; (2) the ‘awareness gap’, characterized by low recognition of CVD risk, particularly the possibility of silent/asymptomatic CVD, uncertainty about lipid (HDL/LDL) targets, and compounded risk when HTN and T2DM coexist, with priority gaps among individuals with lower education, no family history of CVD, or high physical activity; and (3) the proposed primary care solution, in which a brief ‘CVD risk minute’ is integrated into routine HTN/T2DM consultations to explain silent CVD, explicitly link HTN/T2DM to heart attacks and strokes, and discuss cholesterol management and targets as part of risk factor control. Adapted from the open-source tool GAAbstract (https://gaabstract.com/, accessed on 27 May 2026).

The observed disconnect between relatively good knowledge of hypertension and diabetes management and very limited awareness of cardiovascular risk has direct implications for everyday primary care practice in Saudi Arabia. In this cohort, routine contact with the health system among individuals with HTN and/or T2DM appears to have delivered core messages on medication use, blood pressure, and glucose monitoring, along with some lifestyle advice. However, it has not consistently conveyed that CVD can progress silently, that HTN and T2DM act synergistically, or that dyslipidemia is a central component of risk. Figure 4 summarizes this “missing step” by depicting how usual HTN/T2DM care can be extended to include a brief but explicit cardiovascular risk component within the same consultation.

In practical terms, primary care physicians and nurses could incorporate a short “CVD risk minute” into chronic disease visits, during which they (i) state clearly that heart disease and stroke may occur without warning symptoms, (ii) link the patient’s HTN and/or T2DM to future CVD events, and (iii) briefly explain the role of cholesterol, particularly HDL and LDL, using simple language. Visual tools such as color-coded lipid printouts, simple risk charts, or one-to-two-item understanding checks (e.g., “Can heart disease progress without symptoms?”) can be used without substantially increasing consultation time [55].

From a system perspective, these findings support integrating short, standardized CVD risk education prompts into chronic disease clinic workflows and electronic health records, aligned with national cardiometabolic guidelines and Vision 2030 priorities [11]. Particular attention should be directed towards patients with lower educational attainment and those without a family history of CVD, who in this study were least likely to achieve good awareness despite being clinically at risk. For physically active patients, counseling should explicitly emphasize that, while exercise is strongly protective, it does not replace the need for regular blood pressure, glucose, and lipid evaluation, and for appropriate pharmacological treatment when indicated [56].

### 4.5. Using a Multidimensional View of CVD Risk Awareness in Practice

Seeing CVD risk awareness as multidimensional has direct implications for how clinicians and educators work with patients. Instead of asking a single global question (e.g., “Do you know your heart risk?”), Primary care teams can assess and address specific domains:

1. symptoms and the possibility of silent disease;
2. risk factors and their interactions (HTN + T2DM + lipids); and
3. numeric targets or thresholds for blood pressure and cholesterol.

In practical terms, brief checklists or very short questionnaires based on these domains could be used once or twice a year in HTN/T2DM clinics to identify exactly where a patient’s understanding is weakest (for example, lipids vs. symptoms). Education can then be tailored: some patients may need emphasis on silent CVD, while others may need concrete explanations of HDL/LDL and target values. Over time, such domain-focused, quick assessments and conversations could help shift CVD literacy from a general concept to a set of specific, actionable messages that fit naturally into Saudi primary care practice [57,58].

### 4.6. Implications for Practice and Future Research

Taken together, these findings suggest that CVD risk awareness interventions in Saudi Arabia, particularly in the Northern Border Region, should move beyond general advice and explicitly address asymptomatic disease, lipid targets, and the integrated nature of cardiometabolic risk. Educational materials and counseling should segment CVD literacy into distinct domains (e.g., symptoms, lipid/blood pressure thresholds, global risk scores) and tailor messages to individuals with lower education and without a family history of CVD, who appear most vulnerable to low awareness. Integrating structured, brief CVD risk communication into routine primary care visits for HTN and T2DM could help close the identified gaps.

### 4.7. Strengths and Limitations

This study has several strengths. The sample size (*n* = 458) exceeded the calculated requirement, providing adequate power for subgroup and multivariable analyses. The explicit separation of knowledge into HTN, DM, and CVD subscales allowed granular exploration of domain-specific gaps rather than relying on an undifferentiated total score. In addition, the use of multiple regression approaches with detailed diagnostic checks strengthened the robustness of the analytic findings by addressing multicollinearity and model assumptions.

Several limitations must be considered. First, the cross-sectional design precludes causal inference regarding the relationships among knowledge, awareness, and behavioral or clinical outcomes. Second, recruitment via online platforms likely favored younger, more highly educated individuals, potentially underrepresenting older, less educated, and rural populations. Third, clinical data and diagnoses were self-reported rather than verified from medical records, raising the possibility of misclassification and recall bias. Finally, the CVD risk awareness subscale showed lower internal consistency than the HTN and DM subscales, suggesting that future work should refine item content and structure to capture the multidimensional nature of CVD literacy better. The lower internal consistency of the CVD awareness subscale should be interpreted in light of its intended function and content. The eight items were adapted from the Heart Disease Fact Questionnaire and related instruments and were selected to cover several conceptually distinct aspects of risk, silent/asymptomatic CVD, lipid (HDL/LDL) targets, and compounded risk when HTN and T2DM coexist, rather than a single, narrow dimension. In this context, a modest Cronbach’s α reflects the construct’s heterogeneity rather than measurement error and implies greater random measurement error than for the HTN and DM subscales. As a result, the absolute CVD awareness percentages and category assignments should be viewed as approximate indicators of broad awareness levels, and the observed associations with predictors are likely to be conservative (potentially attenuated) rather than exaggerated. Nonetheless, the subscale demonstrated coherent associations with education, family history, diagnosis status, and HTN/T2DM knowledge, which supports its construct validity as a brief indicator of clinically relevant CVD risk awareness. Future studies should expand and refine the item pool to improve internal consistency while maintaining coverage of these key domains.

## 5. Conclusions

Adults in the Northern Border Region of Saudi Arabia demonstrated moderate overall knowledge of hypertension and type 2 diabetes, but very limited awareness of asymptomatic cardiovascular risk and lipid-related concepts. Awareness was higher among individuals with HTN and/or T2DM, higher educational attainment, and a positive family history of cardiometabolic disease, yet remained suboptimal overall. Cardiovascular prevention strategies in this setting should therefore incorporate brief, structured risk communication into routine primary care, with emphasis on silent CVD progression, lipid targets, and integrated cardiometabolic risk, particularly for less educated individuals and those without a family history of CVD. Clinicians should also explicitly counsel physically active patients that lifestyle measures, while beneficial, do not replace regular risk assessment and optimal control of blood pressure, glycemia, and lipids.

## Figures and Tables

**Table 1:** Sociodemographic and clinical characteristics of study participants (*N* = 458).

Characteristics	*n* (%)	95% CI
Sex		
Female	335 (73.1)	[68.8, 77.1]
Male	123 (26.9)	[22.9, 31.2]
Age Group (years)		
18–25	138 (30.1)	[25.9, 34.3]
26–35	62 (13.5)	[10.4, 16.6]
36–46	139 (30.4)	[26.2, 34.6]
>46	119 (26.0)	[22.0, 30.0]
Education Level		
Illiterate	13 (2.8)	[1.3, 4.3]
Basic	22 (4.8)	[2.8, 6.8]
Secondary	92 (20.1)	[16.4, 23.8]
University+	331 (72.3)	[68.1, 76.5]
Diagnosis Status		
No diagnosis	292 (63.8)	[59.4, 68.2]
HTN only	52 (11.4)	[8.5, 14.3]
T2DM only	55 (12.0)	[9.0, 15.0]
Both (HTN & T2DM)	59 (12.9)	[9.8, 16.0]
Family History (HTN/DM)		
Yes	298 (65.1)	[60.7, 69.5]
No	160 (34.9)	[30.5, 39.3]
Lifestyle		
Smoker	59 (12.9)	[9.8, 16.0]
Low Physical Activity	293 (64.0)	[59.6, 68.4]
Moderate/High	165 (36.0)	[31.7, 40.6]

Data are presented as numbers (*n*) and percentages (%) with 95% confidence interval (CI). HTN, hypertension; T2DM, type 2 diabetes mellitus.

**Table 2:** Descriptive statistics for knowledge domain scores and awareness classification (*N* = 458).

Domain	Mean ± SD	Median	% Score	95% CI
HTN Knowledge (Max 7)	5.12 ± 1.40	5.0 (4–6)	73.2%	[71.1, 75.3]
T2DM Knowledge (Max 7)	4.81 ± 1.60	5.0 (4–6)	68.8%	[66.4, 71.2]
CVD risk Awareness (Max 8)	2.46 ± 1.89	2.0 (1–4)	30.8%	[28.6, 33.0]
Total Score (Max 22)	12.40 ± 4.78	13.0 (9–16)	56.4%	[54.0, 58.8]
Awareness Classification				
Poor (<50%)	152 (33.2)			
Moderate (50–74%)	207 (45.2)			
Good (≥75%)	99 (21.6)			

SD, standard deviation; CI, confidence interval; HTN, hypertension; T2DM, type 2 diabetes mellitus.

**Table 3:** Inferential statistics: group differences in total knowledge score (*n* = 458).

Variable	Test Statistic	*p*-Value	Effect Size
Sex (Female vs. Male)	U = 17,234	0.022 *	d = 0.21
Age Group (4 categories)	H = 10.8	0.013 *	η^2^ = 0.07
Education Level (4 levels)	H = 14.9	0.002 *	η^2^ = 0.09
Family History (Yes vs. No)	U = 17,543	<0.001 *	d = 0.48
Diagnosis Status (4 groups)	H = 10.0	0.018 *	η^2^ = 0.06
Geographic Location	H = 8.9	0.031 *	η^2^ = 0.05
Smoking Status	U = 9876	0.028 *	d = 0.18

* Indicates significance at *p*-value < 0.05.

**Table 4:** Multivariable Regression Models Predicting CVD Risk Awareness.

Variable	Model 1: Ordinal Logistic (Predicting Higher Awareness Category)			Model 2: Binary Logistic (Predicting Good Awareness ≥ 75%)		
	Log-OR (SE)	*p*-Value	Sig.	OR [95% CI]	*p*-Value	Sig.
Female Sex	0.529 (0.235)	0.024	*	—	—	
Age Group	−0.033 (0.111)	0.767		—	—	
Education Level	0.489 (0.134)	<0.001	***	1.72 [1.18, 2.52]	0.005	**
Marital Status	−0.347 (0.235)	0.140		—	—	
Family History	0.647 (0.195)	0.001	**	2.35 [1.41, 3.90]	<0.001	***
Diagnosis Status	0.313 (0.096)	0.001	**	1.40 [1.11, 1.76]	0.004	**
Smoking	0.230 (0.302)	0.446		—	—	
Physical Activity	−0.043 (0.138)	0.755		—	—	
Body Weight	−0.034 (0.125)	0.787		—	—	

OR, odds ratio; SE, standard error; CI, confidence interval. * *p* < 0.05, ** *p* < 0.01, *** *p* < 0.001. Note: For Model 2, only statistically significant predictors are displayed with effect sizes. Non-significant variables from Model 1 were excluded from the final binary model presentation.

## Data Availability

The original contributions presented in this study are included in the article/Supplementary Materials. Further inquiries can be directed to the corresponding author.

## Supplementary Materials

The following supporting information can be downloaded at: https://www.mdpi.com/article/10.3390/diseases14070233/s1, File S1: Questionnaire in English. Figure S1: Item-level performance across HTN knowledge. The dashed line indicates a 50% correctness threshold. CVD awareness items demonstrate consistently low correctness, indicating critical knowledge gaps relative to HTN/DM items.

## Abbreviations

The following abbreviations are used in this manuscript:

HTN	Hypertension
T2DM	Type 2 diabetes mellitus
CVD	Cardiovascular disease
HDL	High-density lipoprotein
CI	Confidence interval
SD	Standard deviation
OR	Odds ratio
OLS	Ordinary least squares
AUC	Area under the receiver operating characteristic curve
SPSS	Statistical Package for the Social Sciences
