# Supplementary material for: Cardiovascular Risk Awareness Among Adults in the Northern Border Region of Saudi Arabia: A Cross-Sectional Study with Emphasis on Hypertension and Type 2 Diabetes
Source: Diseases. 2026 Jun 29;14(7):233. doi: 10.3390/diseases14070233 (PMC13408674; doi:10.3390/diseases14070233)
Supplement: Supplementary file 1 [file diseases-14-00233-s001.zip › Supplementary Figure S1.pdf]

Cardiovascular Risk Awareness Among Adults in the Northern Border Region of Saudi Arabia: A Cross-Sectional Study with Emphasis on Hypertension and Type 2 Diabetes

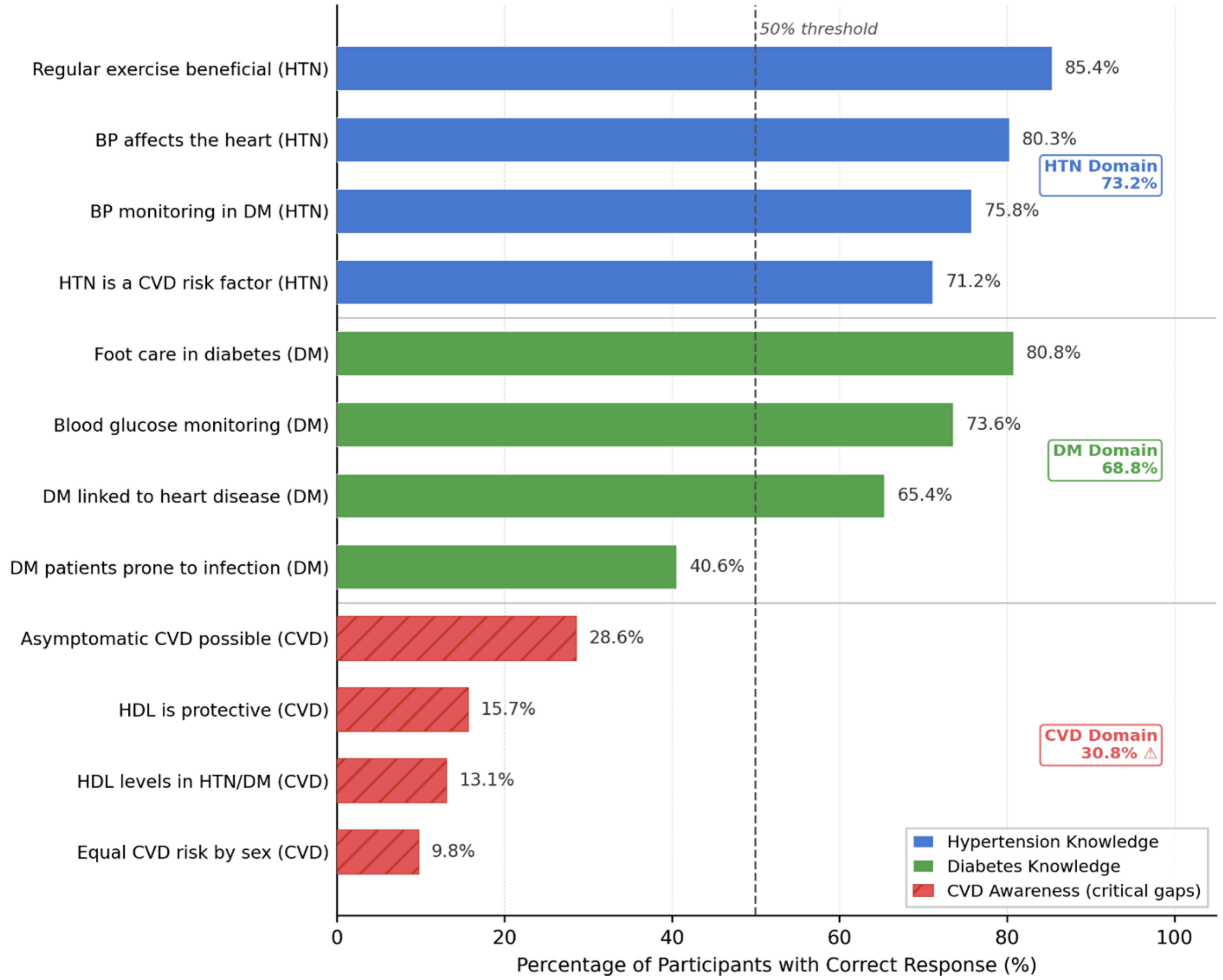

Figure S1: Item-level performance across HTN knowledge, DM knowledge, and CVD awareness domains. The dashed line indicates a 50% correctness threshold. CVD awareness items demonstrate consistently low correctness, indicating critical knowledge gaps relative to HTN/DM items.
