# Supplementary material for: Cardiovascular Risk Awareness Among Adults in the Northern Border Region of Saudi Arabia: A Cross-Sectional Study with Emphasis on Hypertension and Type 2 Diabetes
Source: Diseases. 2026 Jun 29;14(7):233. doi: 10.3390/diseases14070233 (PMC13408674; doi:10.3390/diseases14070233)
Supplement: Supplementary file 1 [file diseases-14-00233-s001.zip › Supplementary File S1.pdf]

Questionnaire in English

A-Personal information

1.	Age<br><br>18-25<br>26-35<br>36-46<br>More than 46
2.	Sex<br><br>Male<br>Female
3.	Weight<br>Less than 50 kg<br>50-69 kg<br>70- 90 kg<br>More than 90
4.	Height<br>Height<br>Less than 150 cm<br>150-169cm<br>170-190 cm<br>More than 190
5.	Nationality<br>Saudi<br>Non-Saudi
6.	Marital state<br><br>Married<br>Single<br>Divorced<br>Widowed
7.	Educational status<br><br>University or higher<br>Secondary school<br>Preparatory school<br>Primary school<br>Illiterate
8.	Occupation<br>• Student<br>An employee of a government company<br>• Employee of a private company<br>• Not working
9.	Level of physical activity<br>Exercise 2−3 times a month or less<br>Exercise 1−3 times a week<br>Exercise 4−7 times a week
10.	Are you a smoker?

	Yes<br>No
11	Family History of Hypertension /Diabetes?<br>Yes<br>No
12	Have you ever been diagnosed with Hypertension?<br>Yes<br>No
13	Have you ever been diagnosed with Diabetes?<br>Yes<br>No
14.	City<br><br>Arar<br>Rafha<br>Turaif<br>Other

B. Hypertension-related characteristics of the study participants in the Northern Border Region, Saudi Arabia:

Duration with hypertension (years)	≤ 2<br>3-5<br>6-9<br>≥ 10
Do you think hypertension is a disease?	Yes<br>No<br>I don't know
Do you think that high blood pressure affects the heart?	Yes<br>No<br>I don't know
Complications of hypertension (Choose more than one)	Heart diseases<br>Renal diseases<br>Eye problem<br>Arterial disease
Do you know normal blood pressure levels?	Yes<br>No
Do you think that A diet rich in salt; a major cause of hypertension?	Yes<br>No<br>I don't know
Do you know the symptoms of hypertension?	Yes<br>No
Do you think that Obesity is associated with hypertension?	Yes<br>No<br>I don't know
Do you think that Regular exercise is beneficial for the control of hypertension?	Yes<br>No

	I don't know

C. Diabetes-related characteristics of the study participants in the Northern Border Region, Saudi Arabia:

How long have you been diagnosed with diabetes?	≥5<br>10–5<br>15–11<br>20–16<br>more than 20<br>Missing
Diabetes regimen	Diet only<br>Oral drugs<br>Insulin injections<br>Oral agents and insulin injections
Do you think that Diabetes can be cured by diet and exercise only?	Yes<br>No<br>I don't know
Do you know the symptoms of diabetes?	Yes<br>No<br>I don't know
Do you think that Obese people are more likely to develop Type 2 diabetes?	Yes<br>No<br>I don't know
Do you think that Type 2 diabetes causes long-term changes to which of the following: nephropathy, retinopathy, arthritis, and digestive disorders?	Yes<br>No<br>I don't know
Do you think that People with diabetes are more prone to infections?	Yes<br>No<br>I don't know
Do you think the Accurate method of monitoring diabetes is accurate?	Yes<br>No<br>I don't know
Do you think that Proper foot care for diabetic patients is important?	Yes<br>No<br>I don't know
Do you think that Blood pressure monitoring is important for diabetic patients?	Yes<br>No<br>I don't know

C. Heart Disease Fact Questionnaire in Northern Border Region, Saudi Arabia:

A person always knows when they have heart disease.	Yes<br>No
If you have a family history of heart disease, you are at risk for developing heart disease.	Yes<br>No<br>I don't know
If your 'good' cholesterol (HDL) is high, you are at risk for heart disease.	Yes<br>No<br>I don't know
People with diabetes rarely have high cholesterol.	Yes<br>No<br>I don't know
People with hypertension rarely have high cholesterol.	Yes<br>No<br>I don't know
People with diabetes or hypertension tend to have low HDL (good) cholesterol.	Yes<br>No<br>I don't know
Men with diabetes have a higher risk of heart disease than women with diabetes.	Yes<br>No<br>I don't know
Feeling weak, lightheaded, or faint is a common symptom of a heart attack	Yes<br>No<br>I don't know
